# Removal of a thoracic intramedullary epidermoid tumor in a child

**DOI:** 10.3171/2023.7.FOCVID2366

**Published:** 2023-10-01

**Authors:** Marianna Di Costanzo, Pietro Spennato, Francesca Vitulli, Maria Allegra Cinalli, Maria De Liso, Claudio Ruggiero, Giuseppe Cinalli

**Affiliations:** 1 Department of Pediatric Neurosurgery, Santobono-Pausilipon Children’s Hospital AORN, Naples;; 2 Division of Neurosurgery, Department of Neurosciences, Reproductive and Odonotostomatological Sciences, Università degli Studi di Napoli "Federico II," Naples;; 3 Department of Medicine and Surgery, University of Milan Bicocca, Milan;; 4 Neurosurgery Department, Ospedale San Gerardo, Monza; and; 5 Department of Pediatric Neuroradiology, Santobono-Pausilipon Children’s Hospital AORN, Naples, Italy

**Keywords:** epidermoid cyst, child, spinal cord, intradural tumor, neurophysiological monitoring

## Abstract

Epidermoid cysts are rare, benign neoplasms that account for less than 1% of all intraspinal tumors. The most common localization is in the lumbar area, and one-third of the tumors are intramedullary. In this video, the authors present removal of a thoracic intramedullary epidermoid tumor in a 6-year-old boy, carrier of a 22q11 gene duplication and affected by psychomotor retardation. He presented a 1-year history of progressive gait impairment. No history of lumbar puncture or trauma was reported. The procedure was performed under neurophysiological monitoring, and it was uneventful with complete recovery of neurological function. Technical nuances are illustrated.

## Transcript

### 0:30 Clinical Presentation.

In this video, we will present the removal of an intramedullary spinal epidermoid tumor in the dorsal spinal cord in a child. The patient is a 6-year-old boy carrier of 22q11.2 gene duplication and was affected by psychomotor retardation. Clinical history was characterized by a 1-year history of progressive gait disturbance until inability to walk alone. Neurological examination at admission showed weakness and mild hypertonia right lower limb, complete right foot dorsiflexion impairment, bilateral hyperreflexia to the lower limbs, and bilateral positive Babinski sign.

### 1:08 Radiology.

MRI showed a large intramedullary tumor at the level of the 10 and 11 thoracic vertebrae. The lesion was heterogeneous in T2 sequences, and there was a restriction of diffusivity in the diffusion sequences. The primary hypothesis was for epidermoid cyst located intramedullary.

### 1:34 Surgical Position and Technical Requirements.

Patient was operated in prone position. Intraoperative neurophysiological monitoring was used. Brilliance amplifier to localize precisely the level was used, and intraoperative microscope of course was used.

### 1:50 Laminotomy.

This is the operative field. We can see the spinous processes. The laminoplasty of three levels was performed, with the piezoelectric device, allowing a very nice laminoplasty.

### 2:00 Opening of the Dura and Dissection of Dural Adhesions.

This is the opening of the dura. The dural adhesions between the dura and the surface of the tumor are easily cut with the Beaver blade. Extensive adhesions are present, but they can be easily cut and separated with the blade. Then the arachnoid is extensively open.

### 2:17 Opening and Dissection of the Arachnoid.

Adhesions between the arachnoid and the surface of the tumor are present, but they can be very nicely removed with the sharp dissection or with dissection with the bipolar forceps. Scissors can be very effective in removing the adhesions between the arachnoid and the surface of the tumor. Inflammatory reaction is very evident in the posterior aspect of the tumor, but the arachnoid finally can be opened very nicely. Standard myelotomy was impossible because the tumor was bulging outside of the spinal cord and was immediately visible below the pial surface.

### 3:10 Capsule Incision.

The dorsal surface of the tumor is now open, with the Beaver blade. The pia was therefore divided on the midline and immediate access to the inner part of the tumor was granted. The small coagulation of the tiny vessels is performed with the bipolar at very, very low power, in order to obtain a perfectly bloodless surgical field.

### 3:37 Removal of Epidermoid Content.

After opening of the tumor, we can remove the epidermoid content very easily with mobilization with a simple Rhoton dissector and the removal either with the tumor forceps or with the simple aspiration cannula. Mobilization is very important before aspiration with the cannula or before removal with the tumor forceps. Here you can see the mobilization of the tumor and aspiration with the aspiration cannula.

### 4:14 Internal Dissection.

It is very easy to perform this internal dissection. There is practically no blood during this part of the procedure. You can see mobilization again of the internal part of the tumor, and this is very important in order to discover the limit between the capsule and the spinal cord parenchyma. Here you can see some remaining part of the internal part of the tumor. We are looking for the deeper part of the tumor.

### 4:49 Removing of the Deep Capsule.

Here we are looking at the parenchyma of the anterior spinal cord. The capsule has been already removed at this stage in this very tiny part, and we try to remove as much as possible of this epidermoid content in order to expose the capsule that is a little bit more difficult to remove if compared to the content of the epidermoid tumor. At this stage, the debulking is extremely satisfactory and we start to remove laterally the remnant of the tumor, and we also see very well the fragment of capsule that are removed together with the content. Here we dissect the fragment of the capsule from the parenchyma of the spinal cord, but they must be removed millimeter by millimeter, with the dissection using either the bipolar forceps or very tiny forceps. It is usually always possible identifying the cleavage plane between the parenchyma and the capsule. At this stage, the depth of the cavity is quite clean, but we have to remove every single fragment, and when adhesions are too strong, it is better to cut than to aspirate inside the aspiration cannula.

6:26 Removal of the Capsule From the Sides of the Lesion. The depth is now clean, and we have to address now the sides of the lesion. We cover and protect the deepest part of the surgical cavity, and we start to remove the fragment of the capsule that are adherent to the lateral aspect of the surgical cavity. At this stage the removal is complete.

### 6:48 Closure of the Spinal Cord and Duraplasty.

We can close the pia of the spinal cord using 7-0 sutures. Three stitches are sufficient in order to limit as much as possible the possible adhesions to the dorsal dura after the closure. This is the surgical field at the end of the closure. A synthetic dural patch was sutured to the dura to avoid adhesions and tethering of the spinal cord. The laminoplasty was secured with reabsorbable stitches, to reduce the risk of postoperative kyphosis.

### 7:25 Pre- and Postoperative Motor Evoked Potentials.

This image shows the motor evoked potentials on the left side and on the right side. The red line represents the motor potentials before beginning of the surgical manipulation of the spinal cord, and the colored line shows the motor potentials at the end of the surgery. As you can see, there is not significant difference comparing the baseline studies and postoperative studies.

### 7:51 Postoperative Course and Imaging.

Postoperative imaging shows complete removal of the tumor both in the sagittal and axial cuts without any remnant inside the cavity of the tumor. The laminoplasty is perfectly in place, as you can see. This is the comparison of preop and postoperative MRI, complete removal, and perfect positioning of the laminoplasty. Surgical operative time was 187 minutes. The postoperative period was uneventful. The patient was discharged at 6 postoperative day. Histological diagnosis was an epidermoid cyst, and no residual tumor was seen on postoperative MRI images. Here we can see the 1-year follow-up MRI showing complete removal of the tumor and the perfect positioning of the laminoplasty.

### 8:43 Conclusion.

The resection of intradural spinal tumors has evolved over the past decade. Preoperative high-resolution MRI allows better preoperative planning.^1–6^ Intraoperative use of neurophysiological monitoring helps the surgeons to preserve patient neurological function, making resection safer and more accurate. Gross-total resection was the goal of surgery to avoid the risk of recurrence and aseptic meningitis. However, if the tumor is tightly attached to the surrounding neural tissue, subtotal excision should be performed to preserve neural function.

## Disclosures

Dr. G. Cinalli reported personal fees from Integra NeuroSciences outside the submitted work.

## Author Contributions

Primary surgeon: G Cinalli. Assistant surgeon: Spennato, Ruggiero. Editing and drafting the video and abstract: Spennato, Di Costanzo, Vitulli, MA Cinalli, G Cinalli. Critically revising the work: Spennato, Ruggiero, G Cinalli. Reviewed submitted version of the work: Spennato. Approved the final version of the work on behalf of all authors: Spennato. Supervision: G Cinalli. Neuroradiologist: De Liso.

## Supplemental Information

### Patient Informed Consent

The necessary patient informed consent was obtained in this study.
